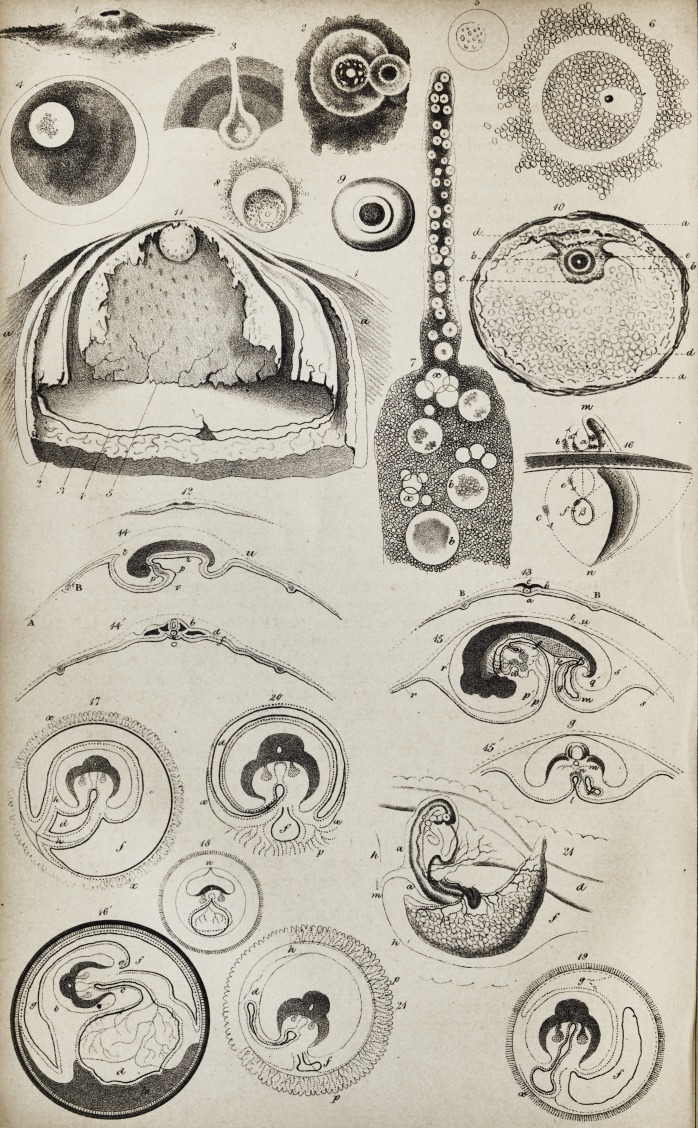# Observations and Reflections on the History of the Development of Animals

**Published:** 1840-01

**Authors:** 


### BRITISH AND FOREIGN MEDICAL REVIEW,

FOR JANUARY, 1840.

### PART FIRST.

### Analytical and Critical Reviews.

### Art. I.

1. *Über Entwickelungsgeschichte der Thiere. Beobachtungen und Reflexionen.* Von Dr. Karl Ernst v. Baer. Erster Theil, mit drei colorirten Kupfertafeln.—*Königsberg*, 1828. 4to, pp. 271.

*Über Entwickelungsgeschichte der Thiere.* Zweiter Theil, mit vier Kupfertafeln.—*Königsberg*, 1837. 4to, pp. 315.

*Observations and Reflections on the History of the Development of Animals.* By Charles Ernest Von Baer. First Part, with three coloured Plates.—*Königsberg*, 1828. 4to, pp. 271.

*On the History of the Development of Animals.* Second Part, with four Plates.—*Königsberg*, 1837. 4to, pp. 315.

2. *Handbuch der Entwickelungsgeschichte des Menschen: mit Vergleichender Rücksicht der Entwickelung der Säugthiere und Vögel: nach fremden und eigenen Beobachtungen.* Von Dr. G. Valentin. —*Berlin*, 1835. 8vo, pp. 658.

*Manual of the History of the Development of Man: with a Comparative View of the Development of Mammalia and Birds: after his own Observations and those of others.* By Dr. G. Valentin.—*Berlin*, 1835. 8vo, pp. 658.

3. *Prodromus Historiæ Generationis Hominis atque Animalium: sistens Icones ad illustrandum Ovi Primitivi, imprimis Vesiculæ Germinativæ et Germinis in Ovario inclusi, genesin atque structuram per omnes animalium Classes multos que Ordines indagatam.* Auctore Rudolpho Wagner, &c. &c. Accedunt Tabulæ duæ æri incisæ.—*Lipsiæ*, 1836. Fol., pp. 15.

*A Precursor of a History of the Generation of Man and Animals: consisting of Drawings illustrative of the Origin and Structure of the primitive Ovum in the Ovary, especially of the germinative Vesicle and Germ; in all the Classes and many of the Orders* By Rudolph Wagner, &c. &c. With two Copper-plates.—*Leipsic*, 1836. Fol., pp. 15.

4. *On the first Changes of the Ova of the Mammifera, in consequence of Impregnation, and on the Mode of Origin of the Chorion.* By Thomas Wharton Jones, Esq. *Communicated by* Richard Owen, Esq. F.R.S. *Phil. Trans.* 1837. pp. 339, 345.

5. *Researches in Embryology. First Series.* By Martin Barry, M.D., F.R.S.E., Fellow of the Royal College of Physicians in Edinburgh. *Communicated by* P. M. Roget, M.D., Sec. R.S. *Phil. Trans.* 1838. pp. 301, 341.

6. *Embryogénie Comparée. Cours sur le Developpement de l'Homme et des Animaux, fait au Museum d'Histoire Naturelle de Paris. Par* M. Coste, *et publié sous les yeux du Professeur par les soins de* MM. Z. Gerbe et V. Meunier. *Avec un Atlas, in 4to, composé de* 20 *Planches, dessinées d'après Nature,* par M. A. Chazal. Tom. I[re].—*Paris,* 1837. 8vo, pp. 477.

*Comparative Embryogeny. A Course of Lectures on the Development of Man and Animals, delivered at the Museum of Natural History of Paris, by* M. Coste, *and published by* MM. Z. Gerbe *and* V. Meunier, *under the Inspection of the Professor. With an Atlas, in 4to, composed of* 20 *Plates, drawn from Nature,* by M. A. Chazal. Vol. I.—*Paris,* 1837. 8vo, pp. 477.

7. *De Organis quæ Respirationi et Nutritioni Fœtus Mammalium inserviunt.* Prolusio Academica, quam scripsit Dan. Fredericus Eschricht, M.D. Professor, &c.—*Hafniæ,* 1837. 4to, pp. 41.

*On the Organs which are subservient to the Respiration and Nutrition of the Fœtus of Mammalia.* By Dan. Frederic Eschricht, M.D. Professor, &c.—*Copenhagen,* 1837. 4to, pp. 41.

The production and the development of organic beings have afforded to philosophers of the greatest talent matter for speculation in all ages. In their endeavours, however, to explain what seemed to the rest of mankind so mysterious, they have been too frequently tempted to assign some external material cause as the effective agent of the process; and so have been lured into the devious paths of imagination, from the less attractive road of onward physiological pursuit. Hence arose the fanciful theories of pre-existence, and of indestructible organic germs; of oxygen and of spermatic animalcules: theories which, even when they had some ground in observed realities, did but remove to a distance, and obscure the difficulties of explanation, without in any degree resolving them.

Modern observations respecting the process of reproduction have been conclusive of two points at least: first, that organic bodies are not preformed; and next, that they are not suddenly produced from formless matter, as by the touch of some magician's wand. Those minute cercariæ, the spermatic animalcules, which were supposed for a time, by Prevôst and Dumas, to penetrate the formless matter of the germ, and so to constitute the first rudiment of the nervous system in vertebrate animals, have themselves supplied to Wagner, Siebold, and Valentin the means of detailing some portion of the history of their gradual development. And whatever simplest form of matter may at any time have been assigned as that upon which vital force, as an external agent, is about to begin its operations—to inform what is rude, to polarize what is indifferent,—yet always, in proportion as the instruments of observation have been perfected, and the zeal of observers has been stimulated by success in similar research, has that matter been shown to possess some organic arrangements, and to perform motions indicative of various energies.

In carefully observing any organism as it seems to come into being, the original primary matter, the secretion of a parent form, is seen to undergo separation and union in such a way that visible distinctions arise in the parts of that which was at first, to all appearance, homogeneous. From single and uniform it becomes multiple and different. The first separation is into solid and fluid, the more solid being still soft, and the more fluid in some degree consistent.* This first change, by which an incomplete antithesis is effected, is not followed by rest; it is but the precursor of others which appear in succession, and which draw new matter within the sphere of their action, derived either from the parent form as new secretions, or from external nature. New matter is constantly taken up, and old matter as constantly laid down. In the mean time, the separation into parts advances—their difference becomes more complete; and, with the new organs thus arising, new activities come into operation. The phenomena alter as each new element is introduced: and the activities with which matter is endowed are seen to change gradually with the gradual change in its form.

Minute anatomy and animal chemistry have not yet advanced to such perfection as to be able to define, satisfactorily, the different changes of structure and composition of matter which accompany the different manifestations of its activity. We know, in the gross, how the composition of muscle differs from that of bone: but we do not know in what respects the composition and arrangement of ultimate particles differ in the same muscle, when in action and when at rest. Yet without such knowledge (to which, indeed, in its perfection we cannot hope to attain,) our insight into the nature of organic processes must necessarily be incomplete. Still we must endeavour to advance it, as best we may, to the utmost; and not, as a refuge from difficulties, be willing to admit that living power is something superadded to dead matter; some distinct entity which, being added, enables the latter to evince those activities which we call vital: unless, indeed, we are content, at the same time, to allow the necessary consequences of that admission, and to confess that physiology is not an experimental science, and that medicine is an impossibility.

The just conclusion, however, appears to be, that the simplest animal matter which we can observe, as, for instance, the first limpid drop in a vesicle of the ovary, is simple only in consequence of the imperfection of our senses, and of the powers with which we aid them. For it performs acts of separation and of composition which are determinate for the species; and the results of these acts are new forms of matter, whose activities, in virtue of their composition, are again determinate: and so another set of consequences, still determinate, arises; and so on, in continual succession, until the creature has fulfilled, by means of its own material productions, the entire series of those acts which make up the idea of its being. Whilst we contemplate these, we cease to distinguish matter from its powers—a living organ from its activities. The change which the matter undergoes is its activity, its action, its power.

* Burdach.

The original matter, then, of any germ, however simple in appearance, has its particles so arranged, its qualities so co-ordinated, that from this arrangement and co-ordination, under the proper external conditions, the whole series of subsequent changes follow each other, step by step, with the utmost order and regularity. The first step involves all the rest: and hence the very simplest form of a germ is said to be *potentially* that animal which it will afterwards *actually* become. In this earliest stage, what is special and peculiar is inappreciable: it is veiled under what is common to the whole animal kingdom, viz. the first appearance of each individual in a fluid form. And when the embryo itself is first visible, whether in vertebrate or invertebrate classes, its form is a primitive streak. But now, though the primite streak be common to all, yet it presents special differences in the vertebrate and invertebrate classes. And this is the character of all subsequent changes which may occur, viz. that what is special (and it may be the precursor of very remarkable distinctions) appears as a mere variety or modification of what is common to large classes or groups. And hence it would seem to be an universal rule, that all varieties of form and structure are only modifications of a form essentially the same throughout the animal kingdom.

It is our wish in this article to lay before our readers the present state of our information respecting the earliest processes of development of the human ovum; for the purpose, not merely of showing how much of that which was lately only inferred from analogy is now actually proved, but, further, with the view of drawing attention to matters of considerable interest upon which enquirers, both in this country and on the continent, are not agreed: such as the origin of the membranes, as they exist in the uterine ovum—the structure of the placenta—the mode of its connexion with the embryo, &c. Yet, though human embryology be the proper end and object of our labours, we do not forget that it cannot be advanced in a right direction, by considering it under any other point of view than as the most elaborate instance of a general process. So that if we were not forced, by the very nature of the case, to supply, by reference to the embryology of birds and mammalia, that which must long be incomplete in the early history of human development, we should still, for the reason mentioned, point to the relations and differences in the corresponding processes which prevail in these different classes.

The papers of Mr. Wharton Jones, and of Dr. Barry, relate to changes of the ovum in the ovary and tube.

The bird's ovary consists of a parenchymatous cellular structure, supplied with vessels, &c., and surrounded by peritoneum, containing multitudes of vesicles which are to become the calices of yelks. In proportion to the vesicles the parenchyme is inconsiderable, so that the ovary has a botruoïdal aspect. In mammalia the parenchyme of the ovary is proportionally much more abundant: towards the surface it assumes more of a fibrous character, constituting the albuginea, and is, as in the instance of the bird, covered by peritoneum. This compact cellular tissue of the ovary, yellowish in colour, is called *stroma*, by Von Baër. It presents round, smooth cavities, in which are lodged the vesicles of De Graaf. Blood-vessels course amongst the fibres of the stroma, and ramify on its cavities.

The first rudiment of an ovum is, as we have said, a minute drop of clear albuminous fluid, secreted by the inner surface of the cavity in the ovary. This fluid drop thickens towards its surface, and thus is an albuminous membrane formed, which is at once a limit between the internal fluid and the walls of the containing cavity, and the medium of their intercourse. Such a separation as this appears to take place more than once, in the fluid contents secreted by the vessels of the stroma; so that, after a time, the ovum within the bird's ovary consists of a fluid sphere and its membrane surrounded by a fluid shell and its membrane. The most internal of the two, viz. the fluid sphere and its membrane, is called, after him who discovered it, the "vesicle of Purkinje," or, generally, the "germ-vesicle;" the spherical fluid shell is called "vitellus," or "yelk;" and its membrane, "vitellary membrane," or "cortical membrane," or "chorion," by different authors. In the smallest ovules, says Valentin, the external yelk-membrane is, with difficulty, separable from the investing membrane of the ovary: it contains a fluid mass, holding suspended a great number of very small round corpuscles, transparent, and only slightly surpassing Brown's corpuscles in size. As the ovule increases in size, the homogeneous transparent mass connecting these bodies shows a consistence as of oil—becomes more tenacious; some round drops, like drops of oil, appear in it, whilst not only these isolated globules, but the whole ovule also, attains a deeper colour. When the ovule has acquired $\frac{1}{6}'''$ in diameter, in addition to these parts, the germ-vesicle, and the granules, and the cicatricula may be distinguished. And this general rule is found to prevail, that the germ-vesicle is largest, proportionally, in the smallest ovules; and in respect of absolute size, is only slightly larger in the most mature ova than in the smallest of the same ovary. In the hen's egg ($\frac{1}{9}'''$ in diameter), the vesicle measures $\frac{1}{20}'''$ (Purkinje).

We have detailed, with some minuteness (after Valentin), these particulars respecting the early ovarian ovule of the bird, that the comparison which he institutes between it and the mature ovarian ovule of mammalia may, hereafter, be intelligible.

The relations of those two important elements of the ovarian ovule—the yelk and the germ-vesicle—were very different from what we have described when first discovered by Purkinje, in 1825. We offer here his description of the envelope of a mature ovarian ovule, and the history of his discovery:

"The inner surface of the yelk-membrane is covered with a delicate and equable layer of globules, about the size of those of the blood, though more transparent; not presenting a fortuitous or irregular mass, but arranged in an organic fashion. Around the cicatricula the globules are more closely crowded, so as to form on all sides a little zone, more than half a line broad, whose inner circuit presents a whitish mammæform cumulus of these same globules, projecting towards the yelk, (see Fig. 1.) On the top of the cumulus is a pellucid pore, visible from either surface of the cicatricula, nearly one sixth of a line in diameter. This pore is never seen in ova of the oviduct or of the uterus: and since I did not find that authors had noted any difference between the cicatricula of the egg and that of the ovarian ovule, I thought it important to investigate the subject further.

"Under a single lens, 1½ inch focal length, I set about destroying the mammæform cumulus in a direction towards its centre, in order to arrive at the pellucid pore. After many attempts, I did but wound a very delicate cuticle, inclosing a limpid fluid. I fancied, rather than possessed any evidence to prove, that there lay something below, of a globular form. Chance at length favoured my numerous and unprofitable endeavours. Whilst removing, by suction, the water in which the object was immersed, the cumulus in question was left dry upon the boss at the bottom of the glass vessel, collapsed and almost dispersed, so that the diameter of the central pore became about double. Whilst examining this with a lens, a most beautiful vesicle, adhering in part to the margin of the pore, in part free, presented itself, to my great admiration. After this, it was not difficult to set it loose. A very minute vesicle, therefore, occupies the pore which seems to perforate the cumulus of the cicatricula; and, being immersed in it, presents only two free surfaces, one towards the outer membrane of the yelk, the other through the top of the little hillock, where it is surrounded, as if by a small crater, towards the interior of the yelk. . . . . Imagination hurried to the conclusion, that this globule might be the germ from which the chick is gradually developed. The next step, therefore, was to investigate carefully the condition of the cicatricula in a recent egg, before incubation, in order to ascertain what changes the vesicle has undergone. It was found that here the cicatricula does not adhere to the yelk-membrane, whilst in the ovarian ovule the two are separated with difficulty: and that in the latter it is readily separable from the subjacent yelk, whilst in the other it adheres thereto pretty closely. The zone of the cicatricula in the ovule, thin and narrow, passes into the cumulus, where the plastic matter appears concentrated. In the cicatricula of the egg, all is extended in breadth, the hillock has disappeared, the semi-pellucid blastoderma is everywhere of the same thickness, and offers not the vestige of a vesicle. The cicatricula now forms a double circle, of which the outer adheres to the yelk, the inner (a continuation of the former) is separated from the yelk by the fossula plana, or, *colliquamentum Malpighii*. This circular fossule resides in the yelk, presents in the middle a whitish umbo (nucleus of Pander), covered with a viscid, semi-pellucid matter, on which white granules, like flour, are sprinkled. (See Fig. 2.) . . . . The vesicle, therefore, of the ovarian ovule has been broken up, and seems changed into the colliquamentum of Malpighi."*

Purkinje is disposed to conclude that the vesicle bursts in consequence of the contractions of the oviduct upon the semi-fluid yelk, as soon as it has passed into the infundibulum—that its lymph is mixed with the substance of the colliculus, and that hence arises the doubly-circular appearance of the cicatricula, &c. The bursting of the vesicle, however, does not depend upon a mechanical cause, as Baër has shown, in his celebrated epistle.

The important vesicle thus discovered by Purkinje, which appears to prepare the cicatricula, after impregnation, for the development of the germ, naturally excited the utmost interest and curiosity. It has, in consequence, notwithstanding the extreme delicacy of the enquiry, been found to exist in every true ovarian ovule in which it has been sought. It was observed by Von Baër, in 1827, in mollusca, annelidæ, crustacea, insects; by Coste, in 1834, in mammalia (rabbit); by Purkinje, Bernhardt, Valentin, and Wagner, in the same year, in mammalia generally; and in this country by T. Wharton Jones, in 1835, in the rabbit. The Prodromus of R. Wagner is especially dedicated to the description of its origin and structure, in all the classes and many of the orders of the animal kingdom. It records, also, an important discovery of his own respecting this vesicle, of which he had previously published a notice, in Froriep's Notizen. If the vesicle be carefully examined under a sufficient magnifying power (a power magnifying 40 or 50 diameters,

* Symbolæ ad ovi avium ante incubationem. pp. 3-5.

for man and the mammalia), there will be found, at some point of its surface, an opaque round spot. This spot Wagner, at first, called macula germinativa. When, however, a higher power was used, he found that the spot is, in reality, a circular flattened layer of extremely minute granules, strictly agglutinated. In mammalia, birds, scaled amphibia, cartilaginous fishes, arachnoïdea, some crustacea, mollusca, conchacea, echinodermata, medusæ, polypi, the macula is almost universally single—and when more than one (which is very rare) there are many maculæ or acervuli, never two, (Figs. 4 & 6). In the batrachia, osseous fishes, some crustacea, the acervuli are numerous, constituting five, ten, or twenty maculæ, (Fig. 5). Hence Wagner now calls this spot "stratum germinativum primitivum." The granules adhere to the interior of the membrane of the vesicle, and are there in contact with its limpid fluid which contains no granules. In proportion as the ovule becomes mature, the connexion between the granules and the membrane of the vesicle is less marked, and the stratum assumes another form. The spot, in those instances where it had been single, spreads out into a number of smaller spots under the surface of the membrane, or even forms a thin nebulous stratum, still more diffused.*

The germ-vesicle, as we have said, at first lies towards the centre of the yelk, and gradually approaches its surface at that point which is most remote from the ovary. Here it is fixed by a layer, more or less circular, of granular matter derived from the yelk, and at length is received into the pore which perforates that layer, (Fig. 3).

The vesicles of the ovary of mammals, which now bear De Graaf's name, had previous to his time been noticed by Vesalius, Fallopius, Bartholin, and others. His contemporaries, also, Van Hoorne and Steno, had described them—by the last of whom they were termed ova—a name adopted by De Graaf, and used by him indifferently with that of vesicle. Valentin, in allusion to De Graaf's work, and in particular to his admirable chapter on the generation of the rabbit, sees reason to infer that probably De Graaf did not consider the vesicles as the ova which are really received into the tubes after impregnation, but that the existence of some smaller ovum, with its proper membrane within the vesicle, was surmised and anticipated, though certainly not demonstrated, by him. We have not received the same impression from De Graaf's work. When he became aware that the ovum in the tube of the rabbit, on the third day, is surpassed in size by the vesicle of the ovary tenfold, he was undoubtedly, as Valentin says, strongly impressed by the fact: but, as we collect from several passages, he accounts for it in another way. His observations of the vesicles in the rabbit, up to the fifty-second hour after copulation, detected no other change than a gradual loss of transparency—an increasing vascularity—their contents becoming more viscid—a papilla on their surface more and more prominent. The observation of the fifty-second hour showed the appearance now called corpus luteum, in four vesicles:† "quibus dissectis," says he, "materiem quasi glandulosam offendimus, in cujus medio exigua cavitas erat, in quâ cum nullum notabilem liquorem compererimus, suspicari cæpimus

* Wagner Prodromus, p. 4, 5. Froriep's Notizen, Nr. 994, p. 53.

† De Mulierum Organis Generationi inservientibus. In Mangeti Bibliothecâ Anatomicâ, i. 478.

num limpida eorum substantia, quæ propriis membranis obvolvitur, disrupta vel expulsa foret." After seventy-two hours, one of the vesicles was found without any fluid; wherefore, on suspicion that an ovum had escaped, it was sought for, and found in the corresponding horn of the uterus, having a size only one tenth of that of the ovarian vesicle: "quod eatenus contingere nobis videtur, quatenus scilicet in testibus (*ovariis*) existentia (*ova* sc.) adhuc aliam materiem complectuntur, illam scilicet, ex quâ glandulosa folliculorum substantia provenit." In another part of his work, alluding to this observation of the rabbit, he says,* "postero die post opacitatem illam conspectam, inter dictas ejus tunicas glandulosam quendam materiem totum ovum involventem globuli figuram repræsentantem intueberis, quæ sensim accrescens ovum undequaque comprimendo, illud tandem per foramen in ejus medio conspicuum expellit: quod in cuniculis tertio post coitum die, &c." It seems clear, from these passages, that De Graaf believed the corpus luteum to be formed between the coats of the vesicle, at the expense (in part at least) of a portion of the fluid contained within its inner tunic, and that the ovum of the tube is, in fact, the diminished vesicle. But this by the way. Many distinguished contemporaries of De Graaf, amongst them Malpighi and Valisnieri, dissented from his opinion that an ovarian ovum is received into the tube. But what more than any other circumstance diverted, for a long period, the attention of physiologists from the search for an ovum in the ovary was, as Valentin remarks, the authority of Haller. After maturely weighing the observations and opinions of his predecessors, he unfortunately came to the conclusion, that the theory of evolution is alone true; that an animalcule escapes from the ruptured ovarian vesicle on conception, so fluid and pellucid as to be invisible, yet still the same which is afterwards seen in the uterus, when its different parts have in some degree been evolved.† Hence the existence of the ovarian ovum, which was strongly suspected by Cruickshank,‡ was not demonstrated until, by the successful labours of Prevôst and Dumas, it was twice observed in vesicles of the ovary after impregnation, as a small spherical body, a millemeter in diameter. Since, however, it was less transparent than the ovule of the tube, they describe it as the ovarian ovule with some hesitation. The honour of discovering the ovum in the *unimpregnated* ovary of a mammal was reserved for Von Baër. He detected it in the dog—presenting an opaque, granulous centre, surrounded by a halo, like the ovum of the tubes (which in the dog he always found to be opaque, when not more than $\frac{1}{15}'''$ in diameter). It was found to vary in size from $\frac{1}{50}'''$ to $\frac{1}{20}'''$, and to be placed in a granular layer and cumulus, within the inner membrane of the Graafian vesicle, exactly as Purkinje's vesicle is placed in the cumulus of the ovarian hen's egg. This cumulus of the vesicle Baër unfortunately considered to be an analogous structure to that of the hen's egg—a mistake into which he was led by the supposed absence of Purkinje's vesicle in the ova of mammals. His view of the mammalian ovum is consequently in some respects exceedingly fanciful, viz. that it is an ovum within an ovum—standing in the relation of Purkinje's vesicle to the ovarian ovule of De Graaf, but in that of the vitellus and its appendages to the fœtus—and presenting, moreover, the strange anomaly of a discus proligerus *external* to the vitelline membrane.

* Op. cit. 463. † Haller, El. Phys. viii., 143, 151. ‡ Phil. Trans. Vol. lxxxvii.

In 1834, Coste corrected this view of Von Baër, and published his discovery of the germ-vesicle in the ovum of the rabbit—thus clearly establishing the general analogy between the ovarian ovum of mammalia and of birds.

The ovum of mammalia touches the inner membrane of the Graafian vesicle at that part which is nearest to the surface of the ovary, and is sustained in its position by a continuation below it of the granular layer called by Von Baër discus proligerus. But the cup which thus contains the ovum (the cumulus of the disc) is larger than it. There is a transparent shell between the two, a space which may perhaps be filled with a clear fluid. When extracted from this, the ovum of mammalia is found to consist, according to Valentin, of—1, an external membrane; 2, a granular layer beneath the former; 3, a contained semifluid, perfectly transparent; and 4, the germ-vesicle. (*Valentin*, p. 19.)

We shall now, after Valentin, point out some distinctions which prevail between the ovarian ova of birds and of mammalia. The external membrane is without any perceptible inner structure in both, unless we admit, in the case of the bird, a very few intricate fibres, irregularly disposed and scarcely perceptible; it has no organic connexion with the neighbouring parts. The yelk, in the unimpregnated bird's egg, consists of three different parts: (*a*) large, oily, yellow, or yellowish yelk-globules; (*b*) very small globules dispersed amongst the former; (*c*) a transparent fluid in which they (*a* and *b*) swim. In the mammalian ovum, the following analogous parts are found: (*c*) the clear transparent fluid, which seems to have a more oily consistence; (*b*) corpuscles, partly corresponding in size to the smaller gobules of the bird's egg, partly something larger than these. The large globules (oil-globules, *a*) are altogether absent. In the central cavity of the bird's egg is a peculiar semifluid mass. A fluid, oily and perfectly transparent is also found in the centre of the mammalian ovule. But in the last the central cavity is not nearly so well defined, nay, it is scarcely to be distinguished from the mass containing granules. The germ-vesicle of the bird's egg is sunk in the disc, just as the ovule of mammalia is sunk in the disc of the follicle: in the mammalian ovule, on the contrary, a much smaller portion of the vesicle is covered by the granular contents. The disproportion of germ-vesicle to yelk, in respect of size, is much greater in the bird than in the mammal. In the bird the egg is surrounded by the membrane and substance of the ovary alone; in the mammal not only by the ovarian follicle but by its disc. From this comparison, Valentin concludes that the ovarian ovule of the mammal is altogether unlike the ovarian ovule of the bird, both being mature. If, on the other hand, the comparison be instituted, between the ripe ovarian ovule of the mammal and the unripe ovarian ovule of the bird, then the similarity is most striking. For in its first stage of development, the bird's yelk is of a grayish-white colour; is composed of an internal membrane perfectly transparent and fibreless; its small globules are without a trace of the true large yelk-globules; and the fluid mass, perfectly transparent, connecting the globules, is especially collected in the centre. The germ-vesicle is now much larger in proportion to the ovule than afterwards, and there is no disc. Valentin asks, does not this description apply strikingly, word for word, to the mammal's ovule? and, in reliance on the affirmative answer, concludes generally, that "the ovum of a mammal perfectly resembles the immature ovum of a bird, and essentially differs from the latter as soon as the yelk-globules have appeared in it."

From the greater relative size of the germ-vesicle in the more unripe ovules, it seems not unreasonable to suppose that if an ovule could be observed, at a period not distant from its first formation, it would be found to consist of germ-vesicle alone. Now, R. Wagner, one of the most skilful observers now living, and the discoverer of the germinative stratum of the vesicle, tells us that he has never been able to find that vesicle free in the mammal's ovary; that, even in ova, which did not exceed $\frac{1}{50}'''$ or $\frac{1}{100}'''$, there was always some appearance of yelk and of its investing membrane, which he calls "chorion," the vesicle being half the size of the whole ovum. When, however, he extended his observations to insects the result was different. The germ-vesicles and their spots are that which is first visible in the tubular ovaries of these creatures, the finest hollow filaments holding in their extremities free germ-vesicles, not more than $\frac{1}{100}'''$ in size, and each of them having a spot of $\frac{1}{400}'''$ (fig. 7). He thus greatly strengthened the probability that the germ-vesicle is the part first formed in every ovum. Dr. Barry has, in the paper before us, supplied, by direct observation, that which Wagner had inferred analogically. He is enabled to describe in two classes of vertebrata, viz. in the rabbit and in the pigeon, observations similar to those of Wagner in the inferior animals, and therefore confirms the belief of former writers, Burdach, Baër, Wagner, "that the germinal vesicle and its contents constitute throughout the animal kingdom the most primitive portion of the ovum."

Dr. Barry next relates his observations of the genesis of the containing membranes of the ovum in some one of the "myriads of minute vesicles, which give to some parts of the ovary, under the microscope, no mean resemblance to the roe of fishes." The fluid holding the granules which surround the germ-vesicle does not appear to be confined in any proper membrane as yet, the whole reside in a cavity sometimes formed in the stroma, sometimes in the walls of an adjacent Graafian vesicle. Afterwards there is gradually seen to form round the enveloping granules a membrane, which Dr. Barry calls the ovisac. It lies loose and unconnected with the walls of the containing cavity, is perfectly transparent, of considerable thickness (as much as $\frac{1}{12}$ of the diameter of the sphere which it bounds), distensible, elastic, apparently unorganized.

Changes now take place in those peculiar granules; the yelk begins to be formed around the germ-vesicle, and is itself now found to be surrounded by *two* membranes, "one the proper membrane of the yelk, membrana vitelli," the other, more external, is called by Dr. Barry the "true chorion." Dr. Barry adds, "the ovum is seen with great distinctness through the transparent membrane of the ovisac, and it is thus possible to follow its different stages of formation." "Subsequently a covering or tunic, consisting of a kind of dense cellular tissue, susceptible of becoming highly vascular and closely connected with the surrounding stroma, is gradually formed upon the outer surface of the ovisac, which, previously in a high degree transparent, now becomes translucent only." (pp. 310-11.)

Dr. Barry apprehends that from the union of this cellular covering with the ovisac, there results what has been called a Graafian vesicle. He extends his examination to the ovisac of amphibia and fishes, and concludes generally, respecting these classes, that the primitive elements of their ova are contained in a vesicle (the "chorion" of authors), essentially the same as that called by him the "ovisac" of mammalia; that the ovisac of mammalia, acquiring a proper tunic, is called a Graafian vesicle, whilst the ovisac of the three other classes acquiring the proper tunic is called a capsule; and further, that, when in these last it becomes pendent from the ovary, and invested by what there is of the substance of the ovary, as well as in some instances by the peritoneum, it is called a calyx. Hence, Dr. Barry clearly shows, that his Graafian vesicle is neither a structure peculiar to mammalia, nor is it analogous to the whole calyx of other animals, but to the capsule.

We cannot praise too highly the care and accuracy with which Dr. Barry has conducted these delicate observations. But we beg to state that his idea of what is to be understood by a capsule and what by a Graafian vesicle does not agree with our own. The Graafian vesicle and the bird's yelk are equally products of the stroma of the ovary which surrounds them, and forms their capsule or envelope. The stroma becomes modified by the presence of its own secretion, the fluid of the vesicle, and undergoes changes such that the capsule, which it always forms, does not at all periods consist of the same elements. The capsule is stroma alone, when the ova of all the classes alluded to by Dr. Barry are mere fluid. And when the ovisac has been formed, the capsule is still stroma alone; whilst the follicle of De Graaf, contained in that capsule, is now ovisac and contents. Next, the stroma allows vessels to pass, and they form in its interior a new vascular membrane, which is more granular, and partakes, according to Baër, of the mucous character. And now the capsule consists of, 1, an external membrane, separable only by maceration from the stroma; 2, an internal, more vascular, membrane; whilst its contents (the follicle) consist of 1, Dr. Barry's ovisac, or Valentin's membrana folliculi; 2, Baër's and Barry's membrana granulosa, or Valentin's membrana cumuli (so called because it swells out round the zona pellucida to form the cumulus); 3, the ovum with the fluid that surrounds it. When the follicle approaches the surface of the ovary, the peritoneum is found to cover a portion of the capsule, and so enters partially into its formation. Dr. Barry believes that the two layers of the capsule, as described by Baër, correspond, the external to his proper vascular covering, the internal to his ovisac. But that is not the case. Baër gives good reasons for maintaining what we have stated above respecting the capsule, and of it Dr. Barry's ovisac forms no part. As a part of the follicle this last is extruded, sooner or later, after the capsule has burst; the capsule, as a part of the ovary, remains to resume its original form of stroma. With respect to Dr. Barry's description of the genesis of the ovisac, there is every reason to believe that it is correct. Its existence was established by what Pockels recorded respecting the ovum of the deer; and appears now to be admitted by Valentin, in his Repertorium, (vol. iii., p. 190,) as we have stated above, though it is not described in his earlier Handbuch.

* Muller's Archiv, 1836. p. 193.

In this respect only does Dr. Barry differ from Pockels, that he describes the cellulo-vascular layer as adhering more closely to the ovisac than to the external layer of the capsule. In the deer the membrane marked (4) in Pockels's plate (Fig. 11), and which corresponds to Dr. Barry's ovisac, is a distinct membrane. He tells us that it remains in the ovary of the sheep, goat, &c., for eight days or more after the escape of the ovule as a bladder full of yellow serum, the corpus luteum forming around it.

It seems probable that in different mammalia this outermost covering of De Graaf's vesicle escapes from the ovary at very different periods after the passage of the ovum into the tube: and moreover, that in the same species it may be, from unassignable causes, retained for a longer or a shorter time; or that, from disease of the ovary, it may not be removed at all. These considerations may assist us in explaining how it may happen that writers of great note differ so frequently in their determination of the seat of the corpus luteum, one placing it in the inner stratum of the theca, another between the strata. Doubtless it is always formed in the vascular inner stratum, which may occasionally be seen covered by the membrane which is, under ordinary circumstances, about to be removed.

But to return to Dr. Barry's paper. When the ovum is completely formed, it is placed in the central region of the ovisac, and keeps its situation there for a time in consequence of the equable diffusion of the peculiar granules in the fluid which surrounds it. These granules enter into the formation of several important structures, and therefore are minutely described. They are ellipsoïdal, generally flattened, sometimes nearly round, "presenting with more or less distinctness a nucleus, or, occasionally, even two nuclei in a single granule," varying in size from $\frac{1}{400}'''$ to $\frac{1}{100}'''$ in length, soluble in water. Their form varies in different orders of mammalia in respect of roundness, and in birds is thought by Dr. Barry to be less regular. They are exceedingly transparent, yet "often punctate, which latter appearance seems sometimes to arise from the presence of minute oil-like globules." They sometimes disappear, apparently by liquefaction. On changes occurring in these granules and the containing fluid, three structures are noted by Dr. Barry to arise. One of these has been described by previous authors as the *membrana granulosa;* the other two have not been hitherto described. Of these, Dr. Barry proposes to call one the *tunica granulosa*, the other (an assemblage of structures rather than a single structure) the *retinacula.* The tunica granulosa is a spherical covering surrounding the "thick chorion," concealing the outer line of its contour, and causing it to appear as a "zone," "halo," or "pellucid space." The retinacula are a central mass surrounding the tunica granulosa, and connected with the membrana granulosa by intervening bands. One use of these bands or cords is "to suspend the ovum and retain it in its situation in the fluid of the Graafian vesicle." It is next conveyed to the surface of this vesicle by subsequent changes in these same structures; the granulous cords on one side of the central mass disappearing, whilst those on the other side are shortened until the ovum has reached the periphery of the Graafian vesicle. "And what is very remarkable, and an interesting instance of *design*, the particular part of the periphery of the vesicle, to which the ovum is thus conveyed, is always that directed towards the surface of the ovary." Here the ovum is held against that surface by the action of the cords which remain, and it is either pressed through the membrana granulosa, or else that membrane is removed by the pressure. Thus it is the tunica granulosa of the ovum, and the central portion of the retinacula, which make up the "cumulus" of Baër, and the band-like portions of the same structure which make up his "flat disc." (Fig. 10.)

The contents, therefore, of a mature Graafian vesicle, will be as follows, according to Dr. Barry:

1, Membrana granulosa, containing fluid, in which are,
2, Tunica granulosa and retinacula (the cumulus and disc of Baër);
3, Chorion ("pellucid zone" or "halo" of other authors);
4, Membrana vitelli;
5, Yelk;
6, Germ-vesicle, with Wagner's granules and its fluid.

We have already shown that Dr. Barry's ovisac ought to head the above list.

These observations of Dr. Barry are exceedingly interesting: they will doubtless be scrutinized strictly by those most capable of verifying or of correcting them. They impress upon us, from some little experience, the conviction that, before any beginner in such pursuits can venture to give an opinion concerning the truth of the results stated, he must well practise his eye to observe the ovum of any animal or class of animals at all periods of its ovarian development, otherwise he will certainly describe what he sees by wrong appellations. For we find that the external membrane of the ovum at one early period is not the external membrane of the next period. That membrane which is called "chorion" by some writers, is a future element of the Graafian vesicle, or capsule according to Barry; whilst his "true chorion," which from the name might be presumed to be really the outermost membrane of the ovarian ovum, is found still to be surrounded by another, viz. the tunica granulosa. The name "zona pellucida" is much less exceptionable than that of "true chorion;" it implies no theory, and certainly it has not yet been shown that any one element of the uterine chorion exists in the ovarian ovum.

Let us now see what other writers say respecting some of the above constituents of the ovarian ovum, which afford the subject of what is presumed to be new in Dr. Barry's paper.

It appears to us that Dr. Barry is original in his description of the tunica granulosa and retinacula; and in his record of the important offices performed by these structures as seen by himself. The existence of Baër's cumulus as a proper envelope of the ovum, has never before, as far as we know, been hinted at; neither has the necessity of accounting for the motion of the ovum from the centre to the surface of the Graafian vesicle been sufficiently obvious to others.

With respect to Dr. Barry's "true chorion," we have seen above, that Valentin describes the ovum as lying in a cup of the disc surrounded by a clear fluid which appears to connect the two intimately by its viscous nature. For he adds (p. 18), that he has never examined the ovum without perceiving traces of the disc. And again, in Müller's Archiv for 1838, p. 534, Valentin states, that when the ovum is found in the centre of the follicle, it is not surrounded by a zona pellucida, but that this zone comes into view as the more fluid contents of the follicle accumulate at the centre, and as the ovum at the same time moves towards the surface; and that it then seems to be relatively broader than it is afterwards. In his Repertorium for 1838, p. 190, Valentin tells us that, according to his experience, the zone is not a transparent mass included in any proper membrane.

Krause, in Müller's Archiv for 1837, p. 27, describes the zone as surrounded by a proper very delicate membrane, which becomes visible when the surrounding granules are removed; and he considers it to be an albuminous fluid confined by that membrane (Fig. 8).

Wagner describes as chorion, in the ovum of mammalia, the same thick pellucid matter as Dr. Barry. (*Prodromus*, p. 11.)

With respect to the vitellary membrane, we are assured of its existence by Valentin and Krause, as well as Barry. Krause describes it as of uniform but small thickness, with well-defined double boundary.

On the other hand, according to Coste and Mr. Wharton Jones, there is only one membrane external to the spherical granular yelk in the ovarian ovum of mammals: this corresponds to that clear, thick envelope called "true chorion" by Barry, and "pellucid zone" by Valentin, but they call it "vitellary membrane."

We may add that the opinion of Carus, as far as concerns the zone, agrees with that of Krause. He considers the external membrane of the zone as described by Krause to be "chorion"—the internal to be "vitellary membrane," the intermediate fluid to be analogous to the "white" of the bird's egg—and that there is no other vitellary membrane.

It seems, therefore, that the question of the number of envelopes of the ovarian ovum of mammals is still undetermined. In our view it is important, inasmuch as the origin of the outer portion of the chorion of the uterine ovum is connected with it.

In mammalia the period of dehiscence of the Graafian vesicle is exceedingly variable. In the sheep, the vesicle opens a few hours after impregnation; in the dog, after several days have elapsed, according to Baër; in the deer, according to Pockels, after five months. Mr. Jones's paper relates principally to the change which the membranes of the ovum undergo in the tube. When he observed the ovum of a rabbit taken from the tube on the fourth day after impregnation, he found, "in addition to the component parts of the ovum of the ovary, a thick gelatinous matter surrounding it." (Fig. 9.) This envelope he believes to have its origin in the ovary as an effect of impregnation. "For in the ova of the rabbit . . . . before impregnation the proligerous disc, in which the ovum is imbedded, is observed to be composed of a gelatinous substance interspersed with grains, but as yet there appears no distinctly circumscribed envelope . . . . . In a rabbit, six days after impregnation, no vitellary membrane was to be seen. The gelatinous-looking envelope constituted the only covering of the yelk, which now formed a vesicular blastoderma." Dr. Barry also, speaking of his membrana vitelli in the same animal, informs us that, after impregnation, "just before the ovum leaves the ovary, this membrane, previously so delicately thin, becomes perfectly distinct and very thick; and that

'the chorion,' imbibing fluid into its interior, becomes somewhat distended, so that a minute space is visible between the membrana vitelli and the chorion. This thickening of the proper membrane of the yelk, and distension of the chorion subsequently proceed much farther, as is proved by the state of the ova found in the Fallopian tube. I find also that the membrana vitelli is still visible, and has considerable thickness in minute ova met with in the uterus." We do not attempt to reconcile their discrepancies; but these authors agree in the fact that the membranes of the ovarian ovum undergo important changes whilst the ovum is in the tube; and we shall endeavour to explain what we conceive to be essential in these changes.

On conception, the vascularity of the ovary, tubes, and uterus is exalted, and the secretions of the different surfaces, viz. of that with which the ovum is, or of those with which it is to be, in contact, are consequently altered. These altered secretions are also much increased in quantity by the presence of the ovum itself when it passes along the tube, or is received into the uterus. We have shown that the external membrane of the ovum is not merely an envelope; but further, that it is the medium of communication between what is within it and what is external to it; and that, to this end, it is formed as a concretion on the surface of the fluid secreted by what is afterwards on its exterior. Now, it is most improbable that, in any case, the same membrane which properly fulfils this office in the ovary will fulfil it in the tube;—for it is under different external conditions: consequently it must be changed. But we shall shortly show that, in some instances, that change is so entire, that when the ovum is in the uterus an absolutely new external membrane is formed. We therefore look upon the above observations of Mr. Jones and Dr. Barry, in the instance of the rabbit's ovum, as proving absorption of fluid from without, by the temporary outer membranes of the ovarian ovum, as preparatory to their entire or partial solution and alteration. And this is quite consistent with what is observed in all other processes of development: for, as Valentin remarks, no part is at once laid down such as it is to continue, even when that continuance is to effect only a temporary purpose.

Mr. Jones believes that the groundwork of the chorion is formed in the tube: so also does Valentin; and we collect from a late notice of the proceedings of the Royal Society that Dr. Barry, in a second paper, relinquishes the opinion, maintained in his first, that the thick transparent membrane of the ovarian ovum deserves the name of "true chorion." In fact, we presume it will soon be generally taught that the external membrane of the ovum changes its form according to the nature of the fluid or solid with which it is in contact, and from which it is its office duly to absorb matter for the nutrition of the ovum. It may undergo either a gradual change, or it may be formed de novo. Of the latter we have instances in the sow and sheep; and it seems to depend upon the quantity of fluid secreted around the ovum; for in the dog, where the quantity is small, the change is gradual. Baër informs us (vol. ii. p. 185,) that he has followed the formation of the external membrane in the sow and sheep through all its stages. It is formed anew in the horn of the uterus. In the sow no trace whatever of that which is afterwards the external membrane can be perceived until the thirteenth day; but the yelksacs are separated from each other by large quantities of albuminous fluid, which distends the sacculated portions of the uterus where they are deposited, and of which they are absorbing a portion, and thereby increasing in size. They are not able, however, to absorb the whole of this fluid; and, therefore, as in the bird's egg, a membrane forms around it. On the thirteenth day this membrane is still so tender that it can only be detected by laying the opened uterus in cold water, when the inner surface is seen to be covered by it, as by a thin film. In ova which are two days older, it has acquired so much consistence that the ova may be removed surrounded by it. Baër was unable to observe this process in the dog or rabbit; for in them the yelk is not drawn out in length, as in the former instances, by the contraction and the form of the inner surface of the uterus, but is closely embraced by the uterus, very little fluid being effused around it. But let us hear Von Baër. "The ovum of the dog, arriving in the uterus, has, like all other ova, an external membrane. If this cuticular membrane can properly transmit the secreted albumen, so that the same may be collected within it, of course no other cuticular covering of the albumen will be formed—there is one already. Since, then, a very little albumen is produced for the ova of carnivora and rodentia—and since the uterus of these animals is so formed that it closes upon the ova at an early period, I think it not improbable that the same membrane which the ovum brought with it as cuticular or external, continues to be such. . . . . It is a matter of indifference to the ovum, if I may be allowed the expression, how it comes by its external membrane; whether that membrane which was external should persist as such (as I must still believe it does in the dog), or whether the albuminous fluid effused around it forms for itself a new one." (Vol. ii., p. 187-8.) In the bird it is the office of many different parts to supply the yelk with investments, both fluid and solid, to prepare it for incubation. The more condensed albumen is secreted around it by the first portion of the oviduct, the more fluid by the portion immediately above its isthmus. In the isthmus the membrane of the shell is formed, and in the uterus the shell itself. In the mammalia, also, analogy is borne out by fact, since new albuminous fluids, and new investing membranes, are formed around the yelks in the tubes and in the uterus.

The views of Coste respecting the chorion are very different from those which we conceive to be correct. He says (p. 107) "the vitelline membrane persists during the whole period of development; it is the analogue of that envelope of the fœtus which is known to anatomists under the name of chorion."

"Whatever be its origin," says Baër, "the external membrane of the ovum in mammalia has, under all varieties of form, the same properties as the shell-membrane of birds, except that it is less dry, inasmuch as it is bathed by fluid, and permeable by it. Until joined by a vascular membrane from the ovum at a later period, it is entirely devoid of vessels. It develops villi on those parts of its surface which come in contact with parts of the uterus which are not quite smooth, or with what may cover such parts. When it receives blood-vessels from the ovum they grow into these villi, and such of the villi as do not receive vessels are but slightly developed. Another general circumstance is, that it consists of two laminæ, at least from the moment when the villi are formed, for one lamina proceeds continuously underneath them. Yet it is not the external lamina which alone forms the villi: for that forms merely their surface. Their substance consists of a mass which is gradually collected between the two laminæ, as may be easily seen in the villi of the ovum of ruminants. We may therefore properly recognize three layers, when the villi are more advanced." (Vol. ii. p. 188.)

This account of the compound nature of the external membrane appears to be received by Burdach, but to be rejected by Valentin, who looks upon the separation as altogether artificial. We give the passage from Baër, because it is not impossible that some one or more of the elements of the ovarian ovum may, in his next paper, be followed by Dr. Barry into the structures thus distinguished.

This villous membrane is that external portion of the future chorion which is called *exochorion* by Burdach and others.

Before we can satisfactorily follow the subsequent elaboration of the chorion, it will be necessary to consider the early development of the central portion of the ovum.

We have already given Purkinje's description of the production of the blastoderma, or germ-membrane, consequent upon the bursting of the germ-vesicle in the hen's egg. There it lies, at first, upon a small portion only of the spherical surface of the yelk; but its periphery is soon extended farther. So rapid indeed is the increase, that at the end of the second day, according to Baër, the germ-membrane covers one half of the surface of the yelk, and at the end of the fifth, the whole of it. As it advances, the yelk-membrane becomes more and more clear and thin on its exterior, and gradually disappears—a broad band of the advancing germ-membrane closely adhering to the retreating membrane of the yelk. This slow and gradual formation of the blastoderma in the bird's egg appears to be superseded in that of mammalia. For here it is found surrounding the whole of the minute yelk, as soon as the latter is so transparent, from solution of its granules after the bursting of Purkinje's vesicle, as to admit of observation. It is presumed, therefore, to be vesicular from the beginning, and Coste appears to be the first observer who insisted upon the probability that it is so.

The modern theory of development, as first proposed by Döllinger and Pander, and successively illustrated by Baër, Rathke, Burdach, &c., proceeds upon the separation of the blastoderma into superimposed layers or laminæ. Above, and most extended, is the *serous* layer; below, and least extended, is the *mucous;* between the two, and later in its appearance, is the *vascular layer*. In one or other of these, as distinct primitive forms, there lies concealed that which is essential in the different organs and tissues of which the body is composed, and in virtue of which they admit of being referred to distinct original groups.

On the serous layer arise the organs of animal life—the brain and spinal cord, organs of senses, skin, muscle, tendons, ligaments, cartilage, bone; on the mucous, the organs of vegetative life—the intestinal canal, the lungs, liver, spleen, pancreas and other glands. The heart and vascular system arise from the vascular layer, "if this is to be considered as a separate layer" (Val.) To which division the generative system is to be primarily referred is still undetermined. Rathke says the vascular.

The separation into two layers, serous and mucous, occurs in the bird's egg, about the twelfth hour of incubation. The mucous layer is much more delicate in the central clear part of the blastoderma, the area pellucida, than in the part external thereto. About the twentieth hour, the layer of granules, forming the vascular layer (Pander), appears.

About the fourteenth hour, a mass of globules, loosely connected together in the transverse axis of the egg and centre of the pellucid area, is seen,—Baër's primitive streak: it is formed on the serous layer (Fig. 12).

The globules of the primitive streak next seem to be resolved : and then there is a change of appearances. On the sides of the streak are the two laminæ dorsales, which bound a median furrow; and below this furrow is the *chorda dorsalis*, which is the axis of the future embryo, and origin of the spinal column. That portion of fluid which separates the chorda dorsalis from the laminæ d., is the future cord and brain (Fig. 13). The chorda dorsalis thickens at the fore part, to form the first appearance of skull, and the fluid between the dorsal laminæ is in larger quantity in correspondence with it; so that the central parts of the nervous system, and their coverings, are laid down at the same time, and grow simultaneously. The separation between the spinal cord and brain is a very early one, and is coincident with a bending downwards towards the yelk of the anterior parts of the laminæ dorsales, which defines the limit between skull and column, brain and cord.

These occurrences have been observed in all the other vertebrate classes, more or less distinctly, except the human. Here, in the absence of direct observations, they are supposed to be referrible to the end of the second, or beginning of the third week.

Next follows the closing of the laminæ dorsales over the fluid which is the rudiment of both brain and cord. The brain, therefore, as Valentin remarks, ought not to be considered as growing from one extremity of the cord. At first there is only a single cerebral vesicle; for in the brain, as well as in the cord, granules accumulate first on the periphery, the central parts continuing to be fluid. The single vesicle is then elongated, and next appears constricted in certain regions, so as to form three cells, which communicate. The anterior cell corresponds to the cerebrum; the middle cell to the corpora quadrigemina and neighbouring parts; the posterior cell to the medulla oblongata and neighbouring parts. These three cells have been observed by Baër, in the dog; by Rathke, in the sheep; by Müller and Burdach, in man.

The deposit of granular matter which accompanies the further development of the brain and cord is seen on that side of both which corresponds to the viscera, sooner than on that which corresponds to the spine.

Two other laminæ (laminæ ventrales of Baër) are in the mean time proceeding from the axis of the embryo, one on each side. They grow out laterally, and tend to converge in the median line, as did the dorsal laminæ; but they form a larger curve, and follow a different direction—that is, they converge to meet *below* the axis: and they do so meet, except in the umbilicus (Figs. 14 and 14[1]).

The mucous layer of the blastoderma gradually surrounds the yelk in birds, and at length covers it, in place of the vitellary membrane. In mammalia, like the blastoderma, it is supposed to have surrounded the yelk from the beginning. It soon permits of division into two portions, which communicate. The smaller portion, which is first beneath and then within the laminæ ventrales, the original of the intestinal canal and its appendages and of the lungs, communicates with the rest of the yelk by the constricted intermediate portion, which becomes narrowed into a duct. (See Fig. 15, where the constriction is far advanced.)

When the earliest vascular system has been established upon the yelk, in mammalia, it takes the name of umbilical vesicle, or tunica erythroïdes. In birds, the yelk appears to be almost exclusively for nutrition of the embryo; its vessels probably derive little from intercourse with the atmosphere. In mammalia, on the contrary, nutrition seems to depend, in a very subordinate degree, upon the contents of the yelk-sac, and rather to be conducted by its vessels, as long as they subsist, by processes similar to the respiratory. Hence the bag is never drawn into the body of a mammal, as in birds, though in them the blood-vessels are more largely disposed upon its surface; but, by gradual lengthening of the intermediate tube, the bowel and the yelk-sac become distinct portions of the vegetative part of the germ (see Figs. 19, 20). And then, at various periods in the different families, the connexion ceases. It seems to persist longer, in proportion to the size which the sac attains, for it grows, even in man, for a time; but the duct is never open in any mammal, on its exit from the ovum, as in birds. There is great variety in external form and size of the yelk-sac, in the different families of mammalia. In most instances it deviates greatly from its early spherical form. In the ungulata, it is drawn out into very long thready productions, from either extremity of a wider middle portion, the former disappearing when the chorion is fully formed. In ruminants, even at a very early period, the middle portion of the sac is alone provided with vessels; and these soon disappear. In the hog, this middle portion persists for the first half of gestation. In the carnivora, the yelk-sac slowly changes its spherical form for that of an ellipsoïd, or spindle: it continually absorbs fluid, and therefore attains a remarkable size; it long maintains an open communication with the intestine, and preserves its rich vascular network until the period of birth. In man, the umbilical vesicle is contrasted to the yelk-sac of the other families, in preserving its form nearly spherical, but agrees with many of them in continuing very small (about four lines in diameter), and in ceasing early to take any share in the elaboration of the embryo. According to Velpeau, the ductus omphalo-entericus is obliterated in man in the fifth week. In some mammalia, the vessels persist, even when the duct is shrunk or obliterated, and the sac collapsed. Such is the case in the horse, the vessels being apparent in the eighth month, though the walls of the sac have fallen together in the seventh. In the rodentia, the cavity of the sac is empty in the mid-period of gestation; and its walls have either grown together, or have joined the chorion, on the one hand, and amnion on the other; but the vessels persist, as tunica erythroïdes, until the period of birth. And in fishes, and the higher amphibia, these vessels are the only bond of union between the vesicle and the embryo, in the later periods of fœtal life.*

It now remains that we notice two very important appendages of the serous and mucous laminæ of the germ-membrane, one the amnion, the other the allantoïs, with its vascular layer.

When, towards the centre of the serous layer, the rudiments of the organs of animal life have been laid down, there still remains a peripheral portion, which is extended over the surface of the yelk. Of this last, a

* Baër, ii. 190 et seq.

fold leaves the edges of the yet unclosed ventral laminæ, and leaving also the mucous layer and yelk, arches over the dorsal surface of the embryo, so as to represent a sac whose opening is the edge of the fold (Fig. 14, *t*, *u*). As the fold advances, the opening is constantly narrowed, and at length vanishes in a point, when the two surfaces of the duplicature constitute two distinct membranes (Fig. 15, *t*, *u*). That membrane which is next the embryo, and which includes it, being continuous with the edges of its ventral laminæ, is the amnion; that which is farthest from the embryo, and which passes into the serous lamina of the blastoderma, on the surface of the yelk-sac, is the "false amnion" of Pander, or the "serous covering" of Baër. That this singular process is conducted in birds in the manner thus described is now, we presume, universally admitted. In mammalia, it is effected with much greater rapidity, yet still in the same way: for Baër has observed it in all its stages, in the dog, the sheep, the pig; he has seen the embryo at first quite uncovered—then partially—then completely inclosed. The amnion is found to lie at first more closely upon the embryo of mammalia than of birds; but in both classes the serous covering, or false amnion, is soon separated from it, and includes both yelk and amnion. Dr. Allen Thomson, in his paper on the human ovum,* fully confirms Baër's account of the formation of the amnion in mammalia. Nor ought it to surprise us: we have seen that the central portion of the serous layer sends the laminæ dorsales arching upwards, to form a tube round the elements of the nervous system—and this is only a repetition of that process for the peripheral portion of the same layer, (see Fig. 22.)

Respecting the "serous covering," Baër remarks,

"It has not previously been observed in the ova of mammalia; and yet without a knowledge of it these ova cannot be understood. Thus Cuvier remarks, that in the ovum of the rabbit the external membrane, which he calls chorion, is dissolved. Since however he recognizes, in addition to chorion, only amnion, allantoïs, and yelk, it follows that these sacs in the rabbit must lie free, and without a containing membrane, which is by no means the case (Fig. 20). It seems to me that we should not meet such strange contradictions amongst authors, in regard to the interpretation of the various portions of the human ovum, if this membrane were taken into account, which undergoes change more rapidly than any other, and which, from its passive rather than its active comportment, presents very different appearances in the same ovum, at periods nearly consecutive. In general, it moves from the yelk-sac towards the outer membrane of the ovum, with which it becomes incorporated. But when and how it gains that situation, depends upon other parts which urge it." (*Baer*, vol. ii., p. 192.)

We are surprised to find that M. Coste gives an account of the formation of the amnion, totally different from that of which we conceive the truth to be indisputably established by Baër and others. According to Coste, the serous portion of the blastoderma consists of two layers, of which the external is detached, like a pellicle, to cover the embryo in the shape of amnion, and to be reflexed into its interior in the shape of peritoneum, from whence it passes again outwards, over the surface of the yelk. Coste admits that direct observation cannot establish the stratification of the serous layer which he insists upon. He is aware also, that his description necessarily involves a denial of the existence of the false amnion; for, speaking of the umbilical vesicle, he says, that "it sometimes persists in animals, until the period of birth, and then only

* Edinburgh Med. and Surg. Journal, No. cxl.

serves as a coif for the embryo, with its double envelope, which has led to the belief in a false amnion."

The allantoïs arises on the fore part of the posterior extremity of the mucous layer, which is closing to form the intestine, as a growth of the intestine which proceeds very rapidly. It passes out where the ventral laminæ are still unclosed, in the region of the umbilicus, and in birds and mammalia reaches, either mediately or immediately, the inner surface of the exochorion. (Marked $f^1$, in all the sections from No. 17 to No. 22.) By the constriction of the navel it is separated into two portions, which communicate: that within the body of the embryo is the sacculated urinary bladder, with the urachus or tube of communication. It receives its vessels from the hypogastric, which are spread out as a vascular layer, especially upon that portion of its surface which faces the exochorion. According to Burdach, the vessels form a distinct layer, endochorion. This is certainly the case in pachydermata, Fig. 19, marked (*g*.) They perforate the albuminous matter which generally intervenes on the inner surface of the exochorion, and, reaching the latter membrane, form with it the placenta.

The form of the allantoïs is subject to remarkable differences in the different classes of mammalia. In carnivora, it resembles the corresponding membrane in birds,—passes from right to left over the back of the embryo, and is only prevented returning into itself by the intervention of the yelk-sac, which is to the left, and below. Towards the surface of the ovum, its opposite portions meet, so that there remains only a triangular space towards the centre, occupied by the yelk-sac. In this class the vascular and mucous layers of the allantoïs do not separate (Fig. 17).

In ungulata, the allantoïs grows so little in breadth, that it does not form a double arch over the amnion, but lies by the side of it. In length, however, it increases so remarkably, that, notwithstanding the great length of the ovum at the earliest periods, it bursts the outer membrane at each extremity, and passes out. Here the mucous and vascular layers separate from each other completely, as soon as the allantoïs has reached the albuminous matter on the interior of the exochorion, and the vessels pass through that matter to seek the villi of the external membrane (Fig. 19).

In rodentia, the allantoïs neither arches over the amnion, nor lies beside it, but keeps the situation opposite to it on the ventral surface of the embryo. It is cylindrical, but small when compared with the size it attains in the two former families. Its vascular layer surrounds it, and then leaves it to form the placenta (Fig. 20, *f*).

In man, the allantoïs continues very small, and appears to be active only in the earliest periods. How the vascular layer reaches the exochorion is not known: it may be raised from the allantoïs, as in the ungulata, and resting upon the amnion, more or less, may pass through the albuminous matter, which in that case would be collected between it and the allantoïs: or the allantoïs may itself bear its own vascular layer to the inner surface of the exochorion. Baër inclines to the latter opinion—because, if the process occurred by separation of the vascular from the mucous layer of the allantoïs, then the vascular layer ought to be found, for some weeks at least, resting upon the amnion in the human subject, as it is in the ungulata throughout the whole period of gestation: but it has never been observed by him in that position* (Fig. 21). We

* These statements are abridged from Baër, vol. ii., p. 195.

may state, however, that Burdach, Müller, and Bischoff, believe that the endochorion leaves the allantois as a single lamina, to join the exochorion.

In the valuable tract by Professor Müller, "De ovo humano atque embryone," published at Bonn, in 1830, the author gives the dissections of two ova, presumed to be of the sixth week, and of one presumed to be of the fourth, or thereabout. In one of the former, there was an appearance which concerns the present subject.

"The amnion was reflected towards its own cavity, forming a sheath for the cord; but that sheath was very short, and opened into the gelatinous space between chorion and amnion, at a distance from the spot where those two membranes were connected together, and where the umbilical vessels spread into the chorion. From the sheath of the cord there proceeded, on the one hand, a very slender duct, which ran along the outer surface of the amnion, and terminated in a pretty large umbilical vesicle filled with a thick substance, and presenting blood-vessels still visible; and, on the other hand, the umbilical vessels, still full of blood, proceeded from the sheath to the outer surface of the amnion. These vessels ran a considerable way in the opposite direction, resting closely upon the outer surface of the amnion, until they entered the chorion at that part where it was joined to the amnion. We must distinguish, therefore, between that part of the cord which is inclosed in a sheath—a small part, indeed, in this case—and that external part of it which is not so inclosed, and where the duct of the umbilical vesicle, and the umbilical vessels, proceed in different directions, yet both resting on the outer surface of the amnion, the one to terminate in the umbilical vesicle, the others to ascend to the chorion."

Müller describes the short portion of the cord included in the sheath, as of great thickness, seeming to hold another vesicle, perhaps the allantoïs, in its interior. He found fluid; but, on account of the extreme tenderness of the parts, he could not satisfy himself respecting their exact relations. This dissection would lead us to conclude, that in man the allantoïs is early shrunk, whilst its vascular covering, the endochorion, is especially developed. That vesicle has been found within the cord, in the position where it was sought for by Müller, in cases recorded by Meckel and Albinus.

We have seen that M. Coste represents the vesicular blastoderma as being transformed by constriction into two lobes, inclosed within his vitellary membrane, the amnion being detached as a pellicle or epidermis from the surface of the blastoderma. He continues the same hypothesis with regard to the allantoïs, which, according to his views, arises by another constriction, as a third lobe of the vesicular blastoderma, between the umbilical vesicle and pubis of the embryo. It expands and grows; and as the ventral laminæ of the embryo become closed, there are two vesicles with their pedicles projecting from its interior, viz. the umbilical vesicle and the allantoïs. At length the caudal extremity of the embryo is fixed to the chorion and uterus, by means of the allantoïs and its pedicle, the cephalic extremity hanging free. A spiral motion occurs round the support, and the two pedicles being in contact, are twisted together. The pedicle of the allantoïs, however, is essentially the future cord,* and the allantoïs, enlarged and flattened, the placenta.

We need scarcely say, that we consider this theory as involving great error; as far as we know, we have stated it fairly, though it is only to be collected by reading a large portion of M. Coste's volume. It does

* Coste, 279.

not account for the existence of a common vaginal portion of the cord which, as every one knows, includes the pedicles of both umbilical vesicle and allantoïs : nay, according to it, but for the spiral twisting, the embryo mammal ought to have two navels. These difficulties are necessary consequences of M. Coste's false estimate of the mode in which the amnion is formed. That error constrains him to consider the allantoïs as formed of all the laminæ of the blastoderma, and not of two only (as it really is), the mucous and vascular. We may add, that according to this view, no explanation can be given of an irregular membrane, frequently observed in a more or less perfect state, between amnion and chorion, and which is the false amnion of Pander, or serous covering of Baër; but which, as we have before mentioned, M. Coste rejects altogether.

Some observations of Baër seem to show that the albumen which is collected under the external membrane of the ovum, exerts a peculiar attraction upon the vessels of the allantoïs. In the pig, the larger trunks reach the layer of that substance on the twenty-third day, on an average: but before the next twenty-four hours have elapsed, the entire vascular lamina of the allantoïs is found to rest upon the exochorion and its albuminous layer. The vessels shoot into the villi of that outer membrane, which rapidly increase in size. From this period, the vascular and mucous layers of the allantoïs appear to separate, and the former to attach itself exclusively to the exochorion. And now (i. e. at the end of the fourth week) vascular layer, albuminous layer, false amnion, exochorion, and villi, grow together, and form a single membrane, of which the lately distinct elements can no longer be separated, the vascular chorion (Fig. 19).

The albuminous substance which appears to play so important a part in guiding the vessels of the embryo to the villi of the chorion, has frequently been noted in dissections of early human ova. Thus Müller, in the work just referred to, in his description of one of the ova in the sixth week, says, "Inter amnion et chorion inde ab insertione funiculi umbilicalis spatium magnum intercedit, substantia gelatinosa plenum, filis varie per gelatinosam materiem ab una ad alteram membranam trajicientibus."* And Dr. Allen Thomson, in examining a human ovum, which he considered to be certainly not older than twelve or fourteen days, found that "the space intervening between the outer surface of the umbilical vesicle and the inner surface of the chorion, was occupied by a thin, tenacious web of albuminous filaments, probably formed by coagulation in the spirits."† And again, in an ovum fifteen days old, he found the same reticulated web ;‡ and again in one aged five or six weeks.§ This is the substance which Velpeau and others have supposed, in the human ovum, to be analogous to the allantoïs of animals,—but erroneously: for, when found, the human allantoïs is always either within the vagina of the cord, or lying as a small compressed vesicle between chorion and amnion, with its duct more or less in the interior of the cord. Dr. Allen Thomson's first two ova, however, supply conclusive evidence that Velpeau's "magma reticulé" is not the allantoïs, for in them the embryo was in so early a stage of development, that it lay flat on the surface of the um-

† Edinburgh Medical and Surgical Journal, cxl., p. 129.

‡ Op. cit., p. 132.

§ Op. cit., p. 133.

bilical vesicle, the intestine not yet formed, and without allantoïs or amnion.

There must be a concurrence of many structures, to secure the formation of a placenta. This we learn from Baër's valuable observations of the ova of mammalia, in the second volume of his work. The vessels of the allantoïs are conducted to the outer membranes of the ovum, by the albuminous layer which is collected beneath it. They proceed there, in order that capillary meshes may be formed round the villi which are in near connexion with the uterus; and they dwindle, and finally disappear, where the villi are not found, or do not increase in size. But villi are only formed on the original external membrane of the ovum. When that external membrane is burst, as it is in carnivora, rodentia, &c., by the rapid growth of the allantoïs, or the yelk-sac, no villi are formed upon the membrane which projects through the openings in it. Nor is the presence of villi the only necessary condition for the further development of the fœtal vessels; there must also be near contact with the uterus, and with the vessels of that organ which, in consequence of the gravid state, are also becoming largely developed. Wherever the villi of the mucous surface of the uterus and their vascularity are undergoing increase, there do the villi of the ovum and its vessels grow out to meet them. And the placentæ will have different shapes, in the different classes of mammalia, according to the forms in which the uterine vessels are developed. In the ruminants, the villi and vessels of the uterus are developed in patches, which form what are called cotyledons: the fœtal placentæ in this class are multiplied to meet them; whilst the uterus on the one hand, and the exochorion on the other, is smooth in the spaces between the cotyledons. In carnivora, the placenta is belt-shaped. In pachydermata, it occupies the whole surface of the uterus, &c. &c.

In all the mammalia, either before or after the passage of the ovum into the uterus, there is found to be effused on the surface of that organ, wherever its vascularity has been exalted, a gelatinous or albuminous secretion. In the human subject, this secretion is the decidua of Hunter, about which so much that is unsatisfactory has been written. It was Hunter's opinion that it covers the whole uterus, with the exception of the three openings into its cavity; an opinion supported by Bojanus and R. Lee. On the other hand, by far the greater number of observers, as Lobstein, Moreau, Breschet, Velpeau, Carus, Burns, trace the gelatinous matter into the tubes, and show that it also closes the neck of the uterus. When the ovum is in the uterus, it is found to be surrounded entirely, or almost entirely, by another gelatinous membrane, apparently similar to the former, and which supports the ovum in near connexion with the wall of the uterus—the decidua reflexa. Different writers maintain very opposite opinions respecting the mode of formation of the reflexa, according as they believe the ovum to attain the interior of the decidua through the opening opposite to a tube, or to push away that decidua which closed the tube, and tended to intercept its passage. The second opinion accords best with observation. Dr. Allen Thomson found, in examining the uterus from which the ovum of fifteen days was taken, that the decidua vera was generally one fourth of an inch in thickness. "The cavity of the decidua was occupied by the usual fluid, and from the posterior wall of the decidua vera, there projected forwards the swelling of the decidua reflexa, containing the ovum imbedded in its substance." So that, as we stated in our Number for July last, when reviewing Dr. Meig's work on midwifery, the ovum must be supposed to glide between the decidua and walls of the uterus, and to carry the decidua before it. Bojanus's observations led him to suppose that for a time there is thus left a portion of the surface of the uterus, between the ovum and it, uncovered by decidua vera,—and that this portion subsequently receives a covering, as a new secretion from the uterus, which he called *decidua serotina.* And this is also Baër's view of the matter, in consequence of his own observations. Both Bojanus and Baër represent the uterine vessels as shooting into the decidua serotina, and so forming the maternal portion of the placenta. In Dr. A. Thomson's case, "the decidua adhering to the inner surface of the mucous membrane of the uterus presented, throughout its whole extent, small blood-vessels, which passed from one membrane to the other." It would seem, in this instance, that the soft and ductile decidua vera closed over the ovum, between it and the uterus, after having allowed the ovum to pass on,—and so involved it in its thickness.

M. Coste maintains the opinion that the caduca is formed in the human uterus, as an adventitious product, *after* the descent of the ovum into its cavity, and supposes that, when formed previous to the descent, it may be a cause of abnormal detention of the ovum in the tube. He rejects the reflexa altogether. Ninety pages of M. Coste's volume are dedicated to criticism of the opinions of others on the subject of the decidua. He denies that it is supplied with vessels from the uterus, and speaks of a maternal portion of the placenta as of a mere phantom of the imagination.

Comparative anatomy teaches us that in all mammalia which have the organ, there are two portions of the placenta; one having its vessels from the embryo, the other from the uterus: and that the two orders of vessels do not communicate.

We shall now review shortly the structure of the placenta in different families of mammalia, in order that—having determined which of them most nearly resemble the human—we may judge how far they can guide us in our estimate of the true structure of the human placenta. That structure, as our readers are aware, has been very differently described by different writers of eminence, who all found their opinions upon observation of facts. Dr. W. Hunter says, in his *Anatomical Description of the Gravid Uterus,* when speaking of the maternal and fœtal portions of the placenta, "each of these parts has its peculiar system of arteries and veins, and its peculiar circulation, receiving blood by its arteries, and returning it by its veins; that the circulation of these two parts differs in the following manner:—in the umbilical portion the arteries terminate in the veins by a continuity of canal, whereas in the uterine portion there are intermediate cells into which the arteries terminate, and from which the veins begin." On the other hand, Dr. R. Lee concludes from his observations (as does M. Coste), "that the placenta does not consist of two parts, maternal and fœtal, and that there is no communication between the uterus and placenta by large arteries and veins. The whole of the blood sent to the uterus by the spermatic and hypogastric arteries, except the small portion supplied to its parietes and to the membrana decidua by the inner membrane of the uterus, flows into the uterine veins or sinuses, and, after circulating through them, is returned to the general circulation of the mother by the spermatic and hypogastric veins, *without entering the substance of the placenta.* The deciduous membrane being interposed between the umbilical vessels and the uterus, whatever changes take place in the fœtal blood must result from the indirect exposure of this fluid, as it circulates through the placenta, to the maternal blood in the great uterine sinuses."*

In the mammalia, in all cases, the fœtal portion of the placenta is formed, as we have seen, by the ramifications of the hypogastric vessels in the villi of the exochorion. In ruminants the ramified villi are collected into masses, called cotyledons: and here the maternal cotyledons which grow to meet them are formed principally from the substance of the uterus. In these elevated masses there are branching cavities, which correspond to the form of the fœtal villi, and receive and embrace them. The uterine vessels surround these cavities, and appear (according to Baër) also to penetrate, to a certain extent, a secreted matter on their surface,—the portion which thus becomes vascular of that matter growing to the surface of the maternal cotyledon.

In the sow there are no cotyledons. The surface of the ovum and of the uterus both present minute folds, which are mutually in near contact—a fold of the exochorion being received between two adjacent folds of the uterus, and vice versâ. In the later periods of gestation the uterine folds, always transverse to the axis of the cavity, are connected by many conjugate folds, so as to assume the form of cells. At the same time the fœtal folds, which were from the first somewhat denticulated, are prolonged into villi, which are received into those cells. The corresponding structures are largely supplied with fœtal and maternal capillaries, which nowhere communicate.

Eschricht describes the placenta of a porpus, in which the rugose chorion is studded with villi closely packed together. On the surface, also, of the uterus are rugæ, but less strongly marked,—with interposed cells, into which the villi of the chorion are received. Each surface has its network of capillaries, which nowhere communicate.

In all these families, the capillary network of the fœtal vessels, though brought into near connexion with the capillaries of the mother, are still separated from them by an epithelium and a mucous secretion. These placentæ have further this common character, that the maternal portion continues in the uterus after birth, the fœtal portion only being thrown off; the separation, too, taking place without rupture of vessels, and without hemorrhage.

Of the human placenta, on the contrary, both the fœtal and maternal portion is extruded at birth, and always with considerable hemorrhage, in consequence of rupture of the maternal vessels; which vessels, according to the generally received opinion, are in the decidua. But this is not peculiar to the human placenta; the same thing occurs in monkeys and in the carnivora.

In the human placenta the villi of the exochorion are longer, thinner,

* Phil. Trans. 1832. p. 63.

more ramified, than in other mammalia, and were therefore long supposed to be vessels. But Lobstein and E. H. Weber have shown that each villus is supplied from the umbilical vessels with a small artery terminating in a small vein on the rounded extremity of the villus. The important question then remains—by what are the apices of the villi, which are collected into masses forming the lobules of the fœtal placenta, surrounded? The Hunters taught, as we have seen that the uterine vessels, after passing through the decidua, open into large cells, formed by cellular substance which loosely connects these lobules. The description of E. H. Weber* has been believed very generally by continental writers to approach the truth more nearly. According to him, the large vessels which leave the uterus to pass into the decidua are deprived of all except their innermost tunics, which are found as soft and tender as coagulated lymph. The veins form a network, and have this peculiarity, that they become wider the more deeply they penetrate between the lobules. Thus the veins themselves form cells or sinuses into which the fœtal villi project. The delicate and yielding coat of the vein is borne inwards by each villus pressing upon its exterior, and so is itself the covering of all the villi which compose the fœtal lobules, and which seem to project into its interior.

Professor Eschricht, one of the physicians of the lying-in hospital at Copenhagen, had an opportunity of examining the placenta, in the case of a woman who died in the moment of delivery. The uterus was carefully removed, the placenta being still retained. The directions of Weber were implicitly followed, the umbilical arteries and veins being alone injected, and not the uterine vessels. And the preparation was exhibited to the Royal Medical Society of Copenhagen, in proof of the accuracy of Weber's description: for, as Eschricht then thought, the fœtal villi were in places seen hanging down into the interior of the venous sinuses. On more careful examination, however, when the sinuses and their large branches were laid open, in many of them no villi whatever could be detected; which appeared the more remarkable when their great number was considered. On further scrutiny, those projections which had before been presumed to be villi were found, beyond dispute, to be merely fibrous threads derived from bloody coagula.

The investigations of Baër having shown a considerable resemblance between the placenta of the carnivora and the human, inasmuch as in both classes not merely the fœtal portion, but the uterine also is extruded at birth; the Professor betook himself to the former, in the hope of clearing away the difficulties which seemed to thicken round the subject. In a cat, immediately after death by drowning, the uterine veins were filled with blue, the arteries with white injection, afterwards the umbilical vessels with red. On dissection, the placenta was found almost entirely detached. Still, as not only the external surface of the placenta, in contact with the uterus, was of a blue dye, but also presented intensely blue points and white pendulous threads, it was concluded that the injection had succeeded. And of this there could be no longer any doubt, when on the fœtal surface, besides the red vessels and their

* Hildebrandt's Anatomie, vol. iv., p. 497.

capillaries, another beautiful ramification of vessels, white and blue, was also clearly visible beneath them. The structure of the placenta was then accurately examined. The blue dye on its uterine surface was found to have its seat in a friable and almost grumous mass, resembling the human decidua. Here the uterine veins, or rather their dilatations only, terminated; for intensely blue points still were seen on the blue ground. White points or threads, indicating the uterine arteries, were also seen, on careful examination, on every part of the same ground. When this soft stratum, corresponding to that part of the human decidua which invests the placenta, was removed, the same membrane, though less soft and friable, was found to penetrate the substance of the placenta. This, according as it was viewed in different directions, presented singular interlacings of the red, blue, and white injections, whilst careful examination of any minute portion proved that the fœtal and maternal vessels did not communicate. The red fœtal capillaries adhered to the most minute laminæ, the villi of the chorion, as described by Baër in the last period of gestation. The blue and white network was composed of vessels three or four times larger than the red, and adhered to the thick and soft laminæ which were evidently productions from the membrane covering the uterine surface of the placenta. It is Baër's opinion that the fœtal laminæ are included in these last; and Eschricht believes it to be well founded.

Since the maternal portion of the placenta is formed in ruminants, pachydermata, and cetacea, almost entirely as a growth of the uterus itself, it might perhaps be concluded analogically, that in carnivora also that portion is merely an altered form of the mucous surface of the uterus, and not a new production during the gravid state. And further, this conclusion might appear to be confirmed by the fact that the vascular villi of the cat are invested by the vascular uterine membrane, according to the disposition observed in those families. Eschricht, however, assures us, that when the surface of the uterus, from which the placenta has been detached, is examined, the mucous membrane is still found to cover it: it is thick, soft, and jagged, from rupture of vessels, but has no resemblance to the decidua; "valde rugosa erat (sc. membr. muc.) et mollis quidem sed neque grumosa nec quasi dissoluta, ita ut membranæ placentam investienti haudquaquam esset similis."

M. Flourens, about the year 1836, revived the notion of a direct communication between uterus and fœtus, in certain families of mammalia, by means of considerable vessels.* He was led by his experiments to divide the mammalia, in this respect, into two classes: viz. those which have a vascular communication by means of a single placenta, and those which have a communication merely by contact or adhesion, by means of multiple placentas. The former case he called communication by continuity, the other communication by contiguity. M. Coste shows, from physiological and other considerations, that Flourens must be wrong. It is more satisfactory, however, to find, from Prof. Eschricht's injection and dissection, that the presumed continuity in one important instance resolves itself into contiguity.

The human placenta and that of quadrumana have, as Hunter pointed out in his paper, undoubtedly very remarkable peculiarities; but let us

* Comptes rendus des Séances de l'Académie des Sciences. 1836. p. 170.

not forget the resemblances which they present to some of those which we have been considering. Now the human placenta resembles the feline in being totally ejected at the period of birth, not merely the fœtal portion, as in pachydermata, ruminantia, cetacea, but its uterine portion also. In both, again, the decidua enters between the lobules, conducting vessels of considerable magnitude. And if the form of the decidua be different, as indeed it is, in these two classes, that difference is referrible to the form of the fœtal villi, which in the human exochorion are arborescent, in the feline laminated. But we are at a loss when we attempt to carry the parallel further; for we do not know how far the decidua penetrates into the interior of the human placenta by means of its productions, nor how the uterine vessels are distributed in the interior of that viscus. Of course we presume, with far the greater number of anatomists, that here the decidua does enter between the lobules, and that it conducts vessels of considerable magnitude, and may suspect that the villi of the exochorion, here so delicately subdivided, do not merely form, as M. Coste supposes, a confused agglomeration, a tangled compressed mass to be clapped upon the walls of the uterus.*

As to the depth to which the decidua penetrates the substance of the placenta, the history of the ovum (says Prof. Eschricht) supplies the best answer. All allow that the villi of the chorion do penetrate the decidua; and therefore the decidua, at least in the earliest periods, supplies vaginal coverings for the villi. What reason then is there for supposing that, at a later period, their ramifications are not similarly covered? It may be more difficult, indeed, to demonstrate a covering now so much the thinner as the ramifications are more numerous, and which can, with difficulty only, be seen distinct and separate from the large vessels which traverse it. If villi, however, be examined with a microscope, says the Professor, they will be seen to be covered with a grumous mass which is doubtless a production of the decidua; and adds that he should scarcely have noticed the vaginæ of the feline villi, though pretty thick, had they not been injected.

With respect to the other question as to the mode in which the uterine vessels terminate in the interior of the placenta: they are supposed to degenerate into crypts or sinuses. But this answer is not satisfactory. For when a change takes place in blood conveyed by vessels, the most minute vessels are, in all other cases, those which are most important. Absorption and secretion are performed by capillaries. And if such a network of uterine capillaries has never yet been seen in the human placenta, Dr. Eschricht very pertinently asks whether any observer has hitherto sought for it by the aid of the microscope in a placenta filled with coloured matter from the uterus? He alleges that no sufficient reason has yet been adduced for concluding that there is no system of uterine capillaries in the placenta intermediate between the arteries and veins.

Doubtless there is much difficulty in conceiving such sinuses as Weber has described, so formed as to receive all the infinitely numerous villi of the exochorion projecting pendulously into their interior from behind their coats. Eschricht avers that he has never seen any such appearance

* Coste, p. 150.

as this; but, on the other hand, that he has often seen the villi covered with decidua. He says that a piece of decidua, torn off for examination, seems, under the microscope, to be studded with fibrils which are obviously the villi of the chorion inserted by their apices firmly and deeply into that membrane. It is plain that *these* villi at least have never floated in any venous sinus. But the supposition is scarcely admissible that one set of villi is thus covered with decidual vaginæ, and that another set is disposed of in sinuses.

Professor Owen, in a letter published by Dr. R. Lee in his paper to which we have before referred, when speaking of the uterine veins which appeared to him to be in general closed "by the apposition of the deciduous membrane and placenta," adds: "the decidua is certainly thinner opposite these orifices than elsewhere; and in some places appeared to be wanting, or, adhering to the vein, was torn up with it; but in these cases the minute vessels of the placenta only appeared, and never any indication of a vascular trunk or cell commensurate with the size of the vein whose terminal aperture had been lifted up from the part." In this there is nothing repugnant to the opinion that the smaller vessels and capillaries may be continued from the uterine vessels which, in some instances, are admitted to have traversed the decidua, and in other instances were closed in consequence of the subsidence (as may be supposed) of that soft and flaccid membrane. The microscopic examination of a monkey's uterus and placenta injected, after the manner employed by Eschricht in the cat, immediately after death, by an observer of Professor Owen's accuracy and skill, would doubtless settle the interesting question of the mode in which the uterine vessels are distributed to the substance of the placenta in the quadrumana—and, by a not unfair inference, in the human subject also. This is a matter deserving the consideration of a society with ample means like the Zoological.

With the exception of the marsupialia and monotremata, which appear to have no placenta, all other mammalia are, in respect of that organ, separated by Prof. Eschricht into two classes. In the one there is a noncaducous uterine portion of the placenta, in the other a caducous uterine portion. To the first class belong the cetacea and all the ungulata; amongst the latter the ruminants being distinguished by the singular form of their cotyledons. To the second belong orders having three different types of placenta: 1, Glires; 2, Carnivora; 3, Simia et Homo.

We have now fulfilled the object which we had principally in view in this article: viz. to place before our readers not only the earliest passages in the development of mammalia and of man, but also the conflicting opinions of the latest writers respecting the mode in which the connexion between embryo and mother is effected, and the nature of the connexion. The enquiry has necessarily led us to consider the development of the peripheral rather than of the central portion of the blastoderma. Had we touched upon this latter part of the subject so fully as its importance demands, we must have occupied an unwarrantable extent of our present Number in a single disquisition. With respect to it, we have merely shown how the primitive organs or tubes are formed; and have avoided almost entirely the subsequent *morphological* modifications of these and

1

2

3

4

5

6

7

8

9

10

11

12

13

14

14'

15

15'

16

16'

17

18

19

20

21

21

their *histiological* separations—or, in ordinary language, the production of the several organs and of the tissues of which they are composed. These may well supply us with matter for some future article, involving, as they do, questions which affect the entire foundations upon which physiology is reared as a science: viz., the origin of the blood, the nature of nutritive secretion and assimilation; the mutual action and reaction upon each other of the differently affected products of those separations to which we have alluded, or the functions of organs, and the external conditions under which alone these actions and reactions can be performed. There are other questions also of the deepest general interest connected with this portion of the subject. How does it assist us in our estimate of the relative perfection of animals and of the mode in which we may legitimately imagine them to be developed from one another? What light is reflected by it upon the relations of the different classes of animals to one another; and upon the progressive development of the entire animal kingdom considered as a whole?

The works which we have placed at the head of this article are all of distinguished merit; two of them of surpassing excellence. To rank amongst the great captains of any science is the lot of very few; their true followers may be many. Baër is one of those illustrious men who have founded the science which has engaged us; and Valentin, though not so distinguished for original discovery, is a successful labourer in the same field: one prepared by education, by taste, by talent, not only to investigate and to adorn the regions laid open by his predecessors, but also to advance their conquests. They who would be imbued with the spirit of the science must study the works of these two authors. With respect to M. Coste, our readers will conclude, from remarks which we have introduced into the body of our article, that we cannot speak of his labours in such decided terms of praise. He is not of the same school, but he is well satisfied to be of another; though whenever he differs from those whose conclusions deserve infinitely greater attention than he affords them, he differs only to involve himself in inextricable difficulties. The execution of his plates is very beautiful. Mr. Jones's paper is a very short one; but he is known as the first observer in this country who detected the germ-vesicle and spot in the ovum of a mammal; and this he claims to have done without previous knowledge of M. Coste's earlier discovery of it: and as the first in any country who discovered these objects in the human ovum. We have already expressed our great satisfaction with Dr. Barry's paper; it is an elaborate and ingenious production. We rejoice to see that the number of our countrymen is daily increasing, who, with Owen, Grant, Allen Thomson, and others, are earnestly contending in the same field where Harvey and Needham and the Hunters formerly achieved a lasting renown, with better means and clearer prospects.

### *Description of the Plate.*

Fig. 1. *Purkinje.* The cumulus of the cicatricula facing the yelk: on its top is a small crater, the inner opening of the pore.

Fig. 2. *Id.* The cicatricula of a laid egg: the inner circle of the blastoderma is raised, so that the cavity of the colliquamentum, the naked nucleus, and the powdery granules are seen.

Fig. 3. *Id.* A segment of a hen's yelk which has been divided in a direction from the cicatricula to the centre. Here is a space containing a pale fluid, from whence a canal is continued to the cicatricula. The germ-vesicle passes from the centre to the surface in this canal.

Fig. 4. *Wagner.* The ovule with its yelk, germ-vesicle and germinative stratum of *Aranea* s. *Epeira Diadema.*

Fig. 5. *Id. Astacus fluviatilis.* A very small ovule $\frac{1}{80}'''$ with a germ-vesicle $\frac{1}{150}'''$. The existence of a limpid yelk is determined from the distance between the vesicle and outer membrane alone. The germinative stratum consists of many points.

Fig. 6. *Idem.* The human ovule from a drawing by Valentin. Most external is some granulous matter of the disc : next the pellucid zone, which bounds the yelk-membrane and yelk : *within is the germ-vesicle* (elongated by the compressor), *with its distinct, round, single macula.*

Fig. 7. *Id.* The posterior termination of the oviduct of an insect, *Acheta Campestris:* the attenuated portion is full of germ-vesicles, $\frac{1}{100}'''$ in size. Below this portion the oviduct is suddenly dilated : here the granular yelk appears, not included in any membrane; oil-globules in groups (fig. *x*) are sprinkled about : germ-vesicles (*b b*) having passed out of the narrowed extremity of the oviduct, are seen of increased magnitude ($\frac{1}{50}'''$ to $\frac{1}{30}'''$). As the oviduct is dilated, the ova are matured, the vesicles increase; each of them is soon surrounded by a yelk and outer membrane, and so the ovules become distinct and separate.

Fig. 8. *Krause* (Müller's Archiv, 1837). Ovule of a goat, which has been subjected to pressure under the microscope. During the process the yelk changes its position within the zona pellucida; the membrane of the latter becomes more visible, the granules of the disc being in part detached and removed ; the membrane of the yelk is much thicker, its double boundary is perceptible when magnified 140 times.

Fig. 9. *Jones* (Phil. Trans. 1837). An ovum found in the Fallopian tube of a rabbit the third day after impregnation; magnified forty diameters.

Fig. 10. *Barry* (Phil. Trans. 1838). Ox. The ovum approaching the periphery of the Graafian vesicle (*a*), being conveyed by (*b*) the retinacula. Tunica granulosa (*c*), membrana granulosa (*d*), (*e*) the ovum.

Fig. 11. *Pockels* (Müller's Archiv, 1836). The different strata of the theca and nucleus of the deer. (*a*), Stroma. 1. Peritoneal covering. 2. External stratum of theca. 3. Internal or vascular stratum of theca. 4. Nucleus, ovum, or follicle of De Graaf, its outer membrane. 5. Membrana granulosa.

Figs. 12, 13, 14, 14′, 15, 15′. From Baër. Sections of the chick at early periods of incubation, magnified about three diameters. The yelk membrane is indicated by an interrupted line, the blastoderma by lines of three different kinds, viz., the mucous layer by a thick dark line; the vascular layer by a closely dotted line; the serous layer by a thin clear dark line. The same lines are preserved in the parts into which these layers are transformed. Fig. 12, the serous and mucous layers, with the primitive streak on the former transverse section. Fig. 13, Laminæ dorsales (*b c*). Chorda dorsalis (*a*), (B) limits of the vascular area, transverse section. Fig. 14 and 14′, longitudinal and transverse sections at the end of second day. In the former (A) is the edge of the blastoderma ;

(B), section of terminal vein of vascular area; (*a*), anterior extremity of chorda dorsalis. The embryo, bent downwards towards the yelk at its anterior and posterior extremities, represents a shield or boat, the blastoderma forming a cap for the head and for the tail of the embryo, (*t*) the anterior part of the fold of the amnion, (*p'*) the inversion of the serous layer, and (*p*) of the mucous layer, a considerable space being left between them where the heart (*v w*) is formed on the vascular layer: from the heart proceed branchial arches, which coalesce to form the aorta (*z*). In the latter, or transverse section 14', the dorsal laminæ have met and surround the fluid of the central nervous system, (*b d*) the ventral laminæ, (*o*) the aorta, (*h*) the superior angle of the mesenteric lamina of the vascular layer, (*f*) the lateral fold of the amnion. Fig. 15 and 15'. Sections on the fourth day. *Longitudinal section*, (*d*) anterior extremity of intestinal canal, afterwards mouth, (*l*) rectum, (*m*) allantois with its vascular layer. Of the serous layer, the reflected peripheral portions (*p' q' r' s'*) have met, by the junction of the points (*t*) and (*u*) of the last figure, so that the amnion (*p' r' t u s' q'*) is formed, and Pander's false amnion, or Baër's serous covering (*rtus*) at the same time. (*e*) The respiratory apparatus; (*f*) stomach; (*g k*) the unclosed part of the intestine. The advanced formation of the heart is seen; and the breadth of the vascular layer, interposed between the chorda dorsalis and intestine, which forms the mesentery. The allantoïs is between the amnion and yelk bag. In the transverse section: (*m*) Wolff's bodies, the false kidneys; (*h*) the superior angle of the mesenteric laminæ, which meet and surround the intestine, which is closed except at (*l*), where is the ductus omphalo-entericus; (*p*) the section of the allantoïs; (*g*) the point where the folds of the peripheral portion of the serous layer meet to form the amnion and serous covering of Baër. Fig. 16 is an ideal drawing to show the mode in which the central portion of the blastoderma is transformed from a plane into the embryo: (*a*) chorda dorsalis—from which the particles of the serous layer, at first horizontal, proceed in the direction of arcs, to form tubes, an upper and a lower set (the arrows indicate the directions); (*b*) arc of formation of dorsal laminæ; (*c*) ditto of ventral laminæ; (*d*) of nervous tube; in the vascular and mucous layers (*e*) is the directing arc of vascular tube; (*f*) of mucous tube; ($\alpha$ $\beta$) central line of all the fundamental organs. In each of these tubes there is a central line and a terminal or closing line, both of which lie in the line $\alpha$ $\beta$. The central lines are those nearest to the axis, or chorda dorsalis—the closing lines those farthest from it. Since the primitive laminæ increase from the centre to the perpihery at the same time that each of them curves downwards to form a tube by closing in the central line also, every point which enters into the formation of the tube must follow the curve determined by these two different motions, and indicated by an arrow. (*n' n*) and (*m' m*), would have been the paths described by the points (*n*) and (*m*), which respectively tend to close the lower and upper tubes formed by the serous laminæ, if those tubes had not been formed until the blastoderma had gained all its development horizontally:—but since the tubes are being formed as the blastoderma grows, the paths of *n* and *m* are very different.

The following drawings, also from Baër, show the relation of the peripheral portions of the blastoderma to the embryo and yelk-sac in their productions—the amnion, serous covering, and allantoïs; and the origin of the placenta in different families of mammalia, as compared with the corresponding processes in the bird.

Fig. 16. Section of the bird's ovum about the eighth day; the embryo being, however, drawn more completely in the long axis of the egg than is natural at this date, for the sake of better comparison. The allantoïs (*f*) has not entirely covered the membrane of the shell. Section of the embryo (*a*), of amnion (*b*) : in the interior of the embryo is seen the section of the false kidney and intestine, as in fig. 15, and from the latter the vitelline duct (*c*) passing to the yelk-sac which is entire (*d*). On the yelk-sac are the omphalo-mesenteric vessels and the terminal vein. Section of urachus (*e*) passing into the sac of the allantoïs, of which the external half (*f*) attaches itself to the membrane of the shell, the inner half (*g*), which is the *membrana media* of older writers and the *endochorion* of Dutrochet, surrounds the amnion, and never touches the membrane of the shell. The allantoïs is seen passing from within the embryo on the right side, and not only arching over its back, but also extending in an opposite direction; the former direction is that which is according to rule, but as the sac grows rapidly and is distended by the secretion of the primordial kidneys, it spreads out wherever there is least resistance. It meets with some resistance from the serous covering and albumen. The remains of the serous covering (*hh*) adhere to the yelk-sac beyond the terminal vein, the allantoïs pushing them before it. Beyond the serous covering is the more compact albumen (*i*) still adhering. At (*k*) is seen the membrane of the shell, and around it the section of the shell itself. To assist the comparison of this egg with that of mammalia, a minute space is left between the shell-membrane and shell, in which the villi of the former are seen : this space is not natural.

Fig. 17. Section of the ovum of carnivora : (*d*) is the transverse section of long yelk-sac which lies in a cavity whose formation has not been properly described by any writer previous to Baër. This space is surrounded by thin laminæ (*hh*) which rest on the neighbouring structures. They are the remnant of the serous covering, the part last removed from the yelk-sac : for the vascular area spreads gradually over the entire yelk-sac, and so the serous covering which is then separated from it, is pushed on by the advancing allantoïs. The allantoïs with its vascular layer inserts itself between the yelk-sac and exochorion, and surrounds the embryo with a double covering (its inner and outer half), one on the outside of the amnion, the other on the inside of the exochorion. The mucous and vascular layers are seen to remain inseparably connected throughout the entire allantois, but the vessels of the latter grow into the villi of the exochorion (*xx*), and so form the fœtal portion of the placenta.

Fig. 18. Section of a very young ovum of a carnivore, to show the serous covering in its perfect condition. It still adheres to the amnion at (*n*); the vascular yelk-sac is seen, but the allantoïs is not yet formed. When it is formed, the part of the serous covering which it will remove last, is that which adheres to the part of the yelk-sac most remote from the embryo.

Fig. 19. Section of ovum of pachyderm, pig. The formation of the chorion is not yet complete. The mucous membrane of the allantoïs is here deserted by the vascular membrane ($g$), which passes on the right hand through the albumen to the villi of the exochorion, and on the left hand rests upon the amnion. Vessels also are seen at ($x$) which leave the vascular layer of the allantoïs to pass into the albumen and villi of the exochorion on the left of the embryo. From these sources the highly vascular placenta is formed. The ovum of ruminants is very similar to this.

Fig. 20. Section of ovum of rabbit. The allantoïs is very long, but small, as the section ($f$) shows. There is some little space between it and the vascular layer, which surrounds it and then gives its vessels to the placenta ($p$). The other parts of the ovum are held together by a very thin membrane ($x$), the serous covering, which having adhered to the yelk-sac passes from it to the placenta. The yelk-sac ($d$) here resembles the allantoïs of carnivora.

Fig. 21. An ideal section of the human ovum, to illustrate Baer's notion of the way in which the vascular layer is conveyed by the allantoïs to the albuminous matter and exochorion to form the placenta: yelk-sac or umbilical vesicle ($d$), allantois ($f$), placenta ($p$), serous covering ($h$) still attached to the amnion.

Fig. 22. The ovum of a pig. The drawing is for the purpose of showing that the amnion is really formed as in the bird. In consequence however of the amnion ($a$) lying very close to the back of the embryo, whilst the serous covering ($h$) is raised to a considerable distance from the yelk-sac ($d$), the opening into the space between amnion and embryo is in form of an infundibulum ($m$), and not a mere oval aperture as in the bird (Fig. 14, *tu*).

---

## Figures and Tables

**Figure f1:**